# Supplementary material for: Resolving the full spectrum of human genome variation using Linked-Reads
Source: Genome Res. 2019 Apr;29(4):635–45. doi: 10.1101/gr.234443.118 (PMC6442396; doi:10.1101/gr.234443.118)
Supplement: Supplemental Material [file supp_gr.234443.118_Supplemental_longranger-2.2.zip › longranger-2.2.tar.bz2!/lr2.2-public/lib/stan/cmdstan/stan/doc/api/html/rep__row__vector_8hpp_source.html]

Stan: src/stan/math/rep\_row\_vector.hpp Source File


|  |  |
| --- | --- |
|  | Stan  2.5.0  probability, sampling & optimization |


- Main Page
- Related Pages
- Namespaces
- Classes
- Files

- File List
- File Members

All Classes Namespaces Files Functions Variables Typedefs Enumerations Enumerator Friends Macros Pages

- src
- stan
- math

rep\_row\_vector.hpp

Go to the documentation of this file.

1 #ifndef STAN\_\_MATH\_\_REP\_ROW\_VECTOR\_HPP

2 #define STAN\_\_MATH\_\_REP\_ROW\_VECTOR\_HPP

3

4 #include <boost/math/tools/promotion.hpp>

5 #include <stan/math/error\_handling/check\_nonnegative.hpp>

6 #include <stan/math/matrix/Eigen.hpp>

7

8 namespace stan {

9

10  namespace math {

11

12  template <typename T>

13  inline Eigen::Matrix<typename boost::math::tools::promote\_args<T>::type,

14  1,Eigen::Dynamic>

15  rep\_row\_vector(const T& x, int m) {

16  check\_nonnegative("rep\_row\_vector(%1%)", m,"m", (double\*)0);

17  return Eigen::Matrix<typename boost::math::tools::promote\_args<T>::type,

18  1,Eigen::Dynamic>::Constant(m,x);

19  }

20

21  }

22 }

23

24 #endif

stan::math::rep\_row\_vector

Eigen::Matrix< typename boost::math::tools::promote\_args< T >::type, 1, Eigen::Dynamic > rep\_row\_vector(const T &x, int m)

**Definition:** rep\_row\_vector.hpp:15

check\_nonnegative.hpp

stan::math::check\_nonnegative

bool check\_nonnegative(const char \*function, const T\_y &y, const char \*name, T\_result \*result)

**Definition:** check\_nonnegative.hpp:52

Eigen.hpp


---

[ Stan Home Page ]

*© 2011–2014,
Stan Development Team.*
